# Evaluation of an Integrated Framework for Biodiversity with a New Metric for Functional Dispersion

**DOI:** 10.1371/journal.pone.0105818

**Published:** 2014-08-22

**Authors:** Steven J. Presley, Samuel M. Scheiner, Michael R. Willig

**Affiliations:** 1 Center for Environmental Sciences & Engineering and Department of Ecology & Evolutionary Biology, University of Connecticut, Storrs, Connecticut, United States of America; 2 Division of Environmental Biology, National Science Foundation, Arlington, Virginia, United States of America; Midwestern University & Arizona State University, United States of America

## Abstract

Growing interest in understanding ecological patterns from phylogenetic and functional perspectives has driven the development of metrics that capture variation in evolutionary histories or ecological functions of species. Recently, an integrated framework based on Hill numbers was developed that measures three dimensions of biodiversity based on abundance, phylogeny and function of species. This framework is highly flexible, allowing comparison of those diversity dimensions, including different aspects of a single dimension and their integration into a single measure. The behavior of those metrics with regard to variation in data structure has not been explored in detail, yet is critical for ensuring an appropriate match between the concept and its measurement. We evaluated how each metric responds to particular data structures and developed a new metric for functional biodiversity. The phylogenetic metric is sensitive to variation in the topology of phylogenetic trees, including variation in the relative lengths of basal, internal and terminal branches. In contrast, the functional metric exhibited multiple shortcomings: (1) species that are functionally redundant contribute nothing to functional diversity and (2) a single highly distinct species causes functional diversity to approach the minimum possible value. We introduced an alternative, improved metric based on functional dispersion that solves both of these problems. In addition, the new metric exhibited more desirable behavior when based on multiple traits.

## Introduction

Biodiversity is a multidimensional concept that includes all aspects of biological variation from those associated with genetics to ecosystem processes [1]. Nonetheless, biodiversity often is studied using a species-based approach with regard to one or more dimensions that reflect the number of species and variation among them with respect to abundances, evolutionary histories [2], [3] or functional characteristics [4]–[6]. Ecological and biogeographic research has been dominated by considerations of taxonomic distinctiveness in which interspecific differences between all possible pairs of species are ignored or considered to be equal. Often species richness is used as a proxy for all aspects (e.g. evenness, dominance, dispersion, diversity) and dimensions (e.g. taxonomic, phylogenetic, functional, genetic) of biodiversity. Recently, a consensus has emerged that such taxonomic approaches do not sufficiently capture important biological variation, with functional and phylogenetic dimensions often responding to environmental factors differently than does the taxonomic dimension [2]–[4], [7], [8].

As a result of the growing interest in phylogenetic and functional approaches for studying community ecology, conservation biology and biogeography, new metrics have been developed to estimate phylogenetic biodiversity (reviewed by [9]) or functional biodiversity (reviewed by [10]), or that integrate multiple dimensions into a single measure [6], [11]–[16], with a goal of making meaningful comparisons between dimensions of diversity and between study systems (i.e. taxa or sites). Nonetheless, comparisons have been confounded because many metrics have undefined units, different units or lack conceptual clarity concerning inherent properties [6], [17]. A framework was developed recently [6] that uses a single conceptual approach for measuring biodiversity based on interspecific differences in abundance, phylogeny and function. This approach also permits the construction of homologous integrated metrics that synthesize data with regard to any combination of dimensions. Nonetheless, the behavior of these metrics has not been explored for exemplar or empirical data. Herein, we use exemplar data structures to evaluate the behavior of these metrics with respect to variation in species abundance, phylogeny or function and introduce an alternative metric for functional biodiversity based on functional dispersion, thereby avoiding some of the shortcomings of the original metric [6].

### An integrated framework

Hill numbers [18] provide the basis for an integrated framework for measuring and comparing biodiversity. The original Hill number formulation estimates the effective number of species in an assemblage if individuals were distributed such that all species in the assemblage are equally abundant (i.e. have equal proportional abundances). Scheiner [6] expanded this framework to include proportional phylogenetic divergence and proportional functional distinctiveness. An alternative approach based on Hill numbers exists that integrates abundance and phylogenetic [12] or abundance and functional information [16]; we briefly compare the conceptual bases for these different approaches in the discussion. The use of Hill numbers ensures that all metrics are in the same units (effective number of species) and are on the same scale (number of species, ranging from 1 to S). In addition, Hill numbers have desirable mathematical properties (e.g. the replication principle) that are lacking in entropies such as Shannon diversity, Gini-Simpson index or Rao's quadratic entropy. These properties provide logical and intuitive results, facilitating comparisons among dimensions and studies [6], [12], [16], [19].

The general form of the Hill number metric is:
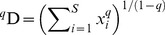
(1)where S is species richness, *x_i_* represents the proportional abundance (*p_i_*), proportional lineage divergence (*l_i_*) or proportional functional distinctiveness (*f_i_*) of species *i* and *q* is a factor (i.e. the order of the diversity metric) that determines how relative proportions are weighted. As *q* increases, species that are more abundant, divergent or distinctive contribute disproportionately more to the magnitude of diversity. Values of 0, 1 or 2 for *q* are common and associated with frequently used metrics: when *q* = 0, ^0^D equals species richness; when *q* = 2, ^2^D is the Gini-Simpson index, also known as the inverse Simpson index [19]. The measure is undefined when *q* = 1, requiring a limit formulation:
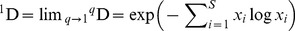
(2)where ^1^D equals the exponential of Shannon diversity.

### Exemplar data

We examined the behavior of metrics from the integrated framework of Scheiner [6] using exemplar data designed to reflect important biological patterns of variation in abundance, phylogeny or function that may occur in assemblages. We begin by examining the independent behavior of each dimension – abundance, phylogeny and function – with most of our effort focusing on the last because it has been the least explored in the literature. These explorations lead to the development of an alternative functional metric. In all analyses, we use *q* = 1 so that species are weighted exactly by their proportional abundance, proportional lineage divergence or proportional functional distinctness. The behavior of Hill numbers for other non-zero values of *q* is qualitatively similar to the behavior when *q* = 1 [16]. Metrics were calculated with script files written in Matlab 7.14.0.739 (available from SJP on request).

### Abundance diversity

Abundance diversity, ^q^D(A), varies as a function of heterogeneity in the relative abundances of species. Exemplar abundance distributions (Table 1) were created to demonstrate maximum diversity (i.e. perfectly even distributions for which all *p_i_* = 1/S), the effect of a single dominant species, the difference between even distributions of rare species compared to even distributions of common species and how randomly generated abundances translate to values of ^1^D(A). (Random numbers were generated via the “rand” function in Microsoft Excel Ver. 14.0.7116.5000.) This type of examination has been previously conducted for ^q^D(A) and is presented here to provide context for similar explorations of phylogenetic and functional diversity.

**Table 1:** Exemplar abundance distributions and resultant abundance diversity values, ^1^D(A).

	Assemblage										
Species	A	B	C	D	E	F	G	H	I	J	K
1	5	100	41	82	91	991	5	11	111	18	95
2	5	100	1	2	1	1	5	11	111	14	74
3	5	100	1	2	1	1	5	11	111	6	60
4	5	100	1	2	1	1	5	11	111	6	34
5	5	100	1	2	1	1	5	11	111	6	26
6	5	100	1	2	1	1	5	11	111	6	14
7	5	100	1	2	1	1	5	11	111	3	6
8	5	100	1	2	1	1	5	11	111	2	5
9	5	100	1	2	1	1	5	11	111	2	4
10	5	100	1	2	1	1	1	1	1	1	2
N	50	1000	9	18	9	9	41	89	889	46	225
^1^D(A)	10.00	10.00	2.38	2.38	1.65	1.07	9.53	9.31	9.05	7.40	6.16

N is the total number of individuals in an assemblage.

^q^D(A) for an assemblage with a perfectly even distribution of individuals among species will always equal S (Table 1; assemblages A and B) for any value of *q*. Because values of ^1^D(A) are based on proportional abundances of species, assemblages in which all common species are equally abundant (assemblages G–I) have greater diversity than do communities in which rare species are equally abundant (assemblages C–F). ^q^D(A) is independent of total abundance. Assemblages with the same proportional abundances (e.g. A and B, C and D) have the same ^1^D(A). Assemblages with one dominant species (assemblages C–F) have low diversity, with ^1^D(A) approaching 1.0 as the proportional abundance of the dominant species increases. Assemblages with randomly generated abundances (assemblages J and K) have intermediate values of ^1^D(A); however, this is partly a result of sample sizes (i.e. ranges of the random values were constrained: 1–20 for assemblage J and 1–100 for assemblage K). Randomly generated abundances that vary more will generally have smaller ^1^D(A) values due to a less even distribution of individuals.

### Phylogenetic diversity

**Figure 1:**
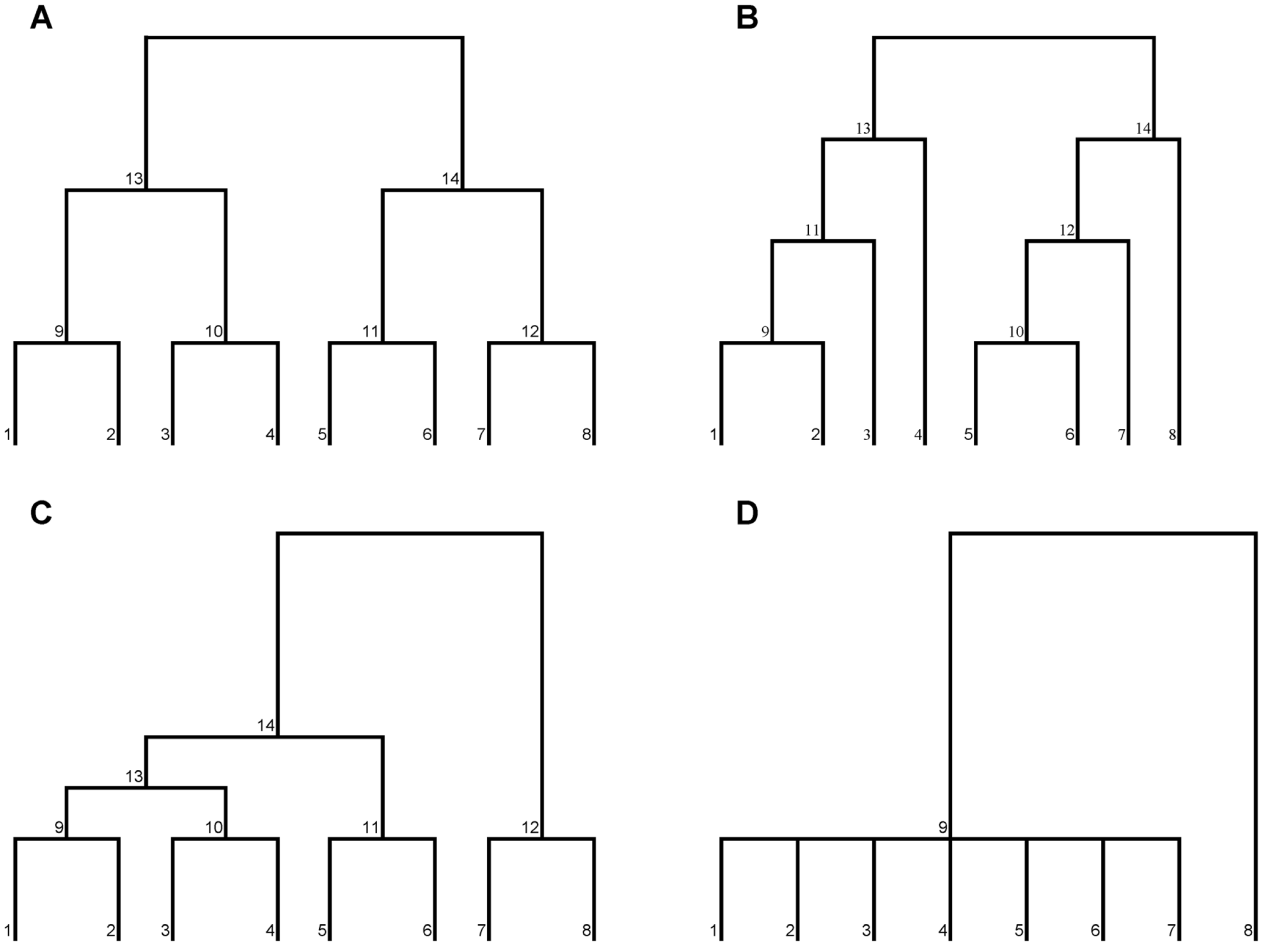
Four cladograms that represent phylogenic relationships among 8 species. Phylogenetic trees differ in the amount and distribution of symmetry. Numbers identify particular branches in each tree, with numbers 1–8 representing tips associated with species. A) A perfectly symmetrical tree. B) A tree that has equally symmetrical basal clades, but that is asymmetrical within each clade. C) A tree that is symmetrical toward the tips, but asymmetrical toward the root of the tree. D) A tree that is symmetrical within the polytomy associated with species 1–7, but that has one distantly related species.

Phylogenetic diversity, ^q^D(P), reflects variation in the proportional lineage divergences of species. The total amount of divergence in a phylogeny (i.e. Faith's PD [20]) is analogous to the total number of individuals in an assemblage and proportional lineage divergences (*l_i_*) are analogous to proportional abundances (*p_i_*). Here we demonstrate the behavior of ^q^D(P) with examples that differ in the topology of the phylogenetic tree, including a perfectly symmetrical tree (Figure 1A), a tree that has two identical clades but that is asymmetrical within each clade (Figure 1B), a tree that is symmetrical toward the terminal branches but that is asymmetrical deeper in evolutionary time (Figure 1C) and a tree that has many closely related species in a polytomy and one distantly related species (Figure 1D). In addition, for each topology, multiple patterns of branch length are explored to demonstrate how variation in basal, terminal and internal branches affect phylogenetic diversity (Table 2). In systematics, symmetry typically refers only to topology. Our definition of symmetry has an additional constraint, requiring both topology and branch lengths to be the same for each branch point of the tree. Consequently, only a tree that is symmetrical throughout evolutionary time (Figure 1A) is perfectly symmetrical by this definition. These differences in topology are also related to the property of “regularity” as defined by Pavoine and Bonsall [8].

**Table 2:** Branch lengths representing time since evolutionary divergence for exemplar phylogenetic trees.

Branch	Tree A				Tree B				Tree C				Tree D			
number	1	2	3	4	1	2	3	4	1	2	3	4	1	2	3	4
1	20	98	1	1	20	92	2	2	20	92	2	2	10	99	1	1
2	20	98	1	1	20	92	2	2	20	92	2	2	10	99	1	1
3	20	98	1	1	40	94	4	49	20	92	2	2	10	99	1	1
4	20	98	1	1	60	96	6	96	20	92	2	2	10	99	1	1
5	20	98	1	1	20	92	2	2	20	92	2	2	10	99	1	1
6	20	98	1	1	20	92	2	2	20	92	2	2	10	99	1	1
7	20	98	1	1	40	94	4	49	20	92	2	2	10	99	1	1
8	20	98	1	1	60	96	6	96	20	92	2	2	100	100	100	1000
9	30	1	1	98	20	2	2	47	10	1	1	46	90	1	99	999
10	30	1	1	98	20	2	2	47	10	1	1	46				
11	30	1	1	98	20	2	2	47	20	2	2	92				
12	30	1	1	98	20	2	2	47	80	8	98	98				
13	50	1	98	1	40	4	94	4	10	1	1	46				
14	50	1	98	1	40	4	94	4	60	6	96	6				
																
Faith's PD	380	790	208	402	440	764	224	494	350	755	215	350	260	794	206	2006
^1^D(P)	8.00	8.00	8.00	8.00	7.88	7.99	7.99	7.50	7.83	7.99	7.12	7.92	7.45	8.00	5.44	5.30

Branch numbers correspond to those in Figure 1. All examples are ultrametric and have a tree height (distance from root to tip) of 100 or 1000 (only for example 4 of tree D). For each tree, branch lengths in example 1 correspond roughly to the scale at which they are drawn in Figure 1, example 2 places most of the evolutionary time in the tips of the trees, example 3 places most of the evolutionary time in the most basal branches, example 4 for tree D further accentuates the amount of evolutionary history placed in the basal branches, and example 4 for trees A, B, and C places most of the evolutionary history in internal branches. Faith's PD [20] equals total lineage divergence (L). ^1^D(P) is phylogenetic diversity.

^q^D(P) has a maximum value equal to species richness when all species in a phylogeny have equal divergences, which occurs when the phylogeny is perfectly symmetrical (Table 2; Figure 1A). Any deviations from perfect symmetry will decrease ^q^D(P), whether asymmetry occurs within clades of a phylogeny (Figure 1B) or exists more basally in the phylogeny (Figure 1C). Values of ^q^D(P) are lowest if a tree has many closely related species and one distantly related species (Figure 1D). The theoretical minimum value for ^q^D(P) is 2.0, as any phylogeny has to have at least two branches; however, it is unlikely that ^1^D(P) would approach 2.0 unless the most distantly related species was extraordinarily distant (Table 2, tree D4). Because ^q^D(P) is based on proportional lineage divergences, the magnitude of the effect of asymmetry in a tree is contingent on the amount of evolutionary time associated with that asymmetry. For example, if branches 1–7 in tree D are long compared to branch 9 (Table 2, tree D2), the tree is highly symmetrical for most of evolutionary time and ^1^D(P) approaches S. In contrast, if branches 1–7 are short compared to branch 9 (Table 2, trees D3 and D4), the tree is highly asymmetrical for most of evolutionary time and ^1^D(P) decreases substantially.

One of the desirable characteristics of Hill numbers is that they obey the replication principle: when combining N equally weighted phylogenetically, functionally or taxonomically distinct assemblages (i.e. assemblages with no lineages, functions or species in common) that have the same diversity X, the pooled assemblages will have 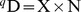 [19]. This framework exhibits the replication principle. For any set of branch lengths for tree A (Figure 1), ^1^D(P)  = 4.0 for each half of the tree and ^1^D(P)  = 8.0 for the entire tree. Similarly, because each half of tree B has the same topology, ^1^D(P) for each half of the tree is the same and ^1^D(P) for the entire assemblage is twice that value. For phylogenetic data, replication holds only when the trees are combined at the root, and species in the two assemblages belong to separate clades. Otherwise, the combined tree will result in new branching points that are interior to each of the original trees and the total lineage length (i.e. Faith's PD) will not be a simple sum of the values for the two original trees. The reason that replication is more restrictive for phylogenetic diversity is that a cladogram includes relational information, whereas abundances do not do so. Changes in relationships necessarily alter the phylogenetic diversity measure. A commonly used approach for phylogenetic diversity is based on Rao's Q, for which a numbers equivalent was developed [21]. However, Rao's Q used this way fails the replication principle [19].

Our framework can be used to evaluate the relative symmetry of different hierarchical levels of a phylogeny by splitting phylogenies into clades and calculating ^q^D(P) for each clade and for the entire tree. The sum of ^q^D(P) for clades from a phylogeny that are similar in symmetry will approach the value of ^q^D(P) for the entire phylogeny. If each clade exhibits greater symmetry than the entire tree, the sum of ^q^D(P) from the clades will be greater than the value for the entire tree. In contrast, if each clade is less symmetrical than the entire tree, the sum of ^q^D(P) from the clades will be less than the value for the entire tree. Other measures of tree symmetry exist [22]–[25]; our suggested usage of ^q^D(P) is not intended to replace those, but to provide a measure that can be used in the context of other dimensions of diversity.

### Functional diversity

Functional diversity, ^q^D(F), measures variation in the functional distinctiveness of species, which Scheiner [6] based on non-overlapping volumes in functional space. Those non-overlapping volumes are calculated from minimum functional distances (i.e. for each species, the distance to the nearest neighbor in functional space). The total unique functional volume is analogous to the total number of individuals in an assemblage for ^q^D(A) and to the total amount of divergence in a phylogeny for ^q^D(P). One important difference is that non-unique functional volumes do not contribute to ^q^D(F), whereas all abundances or branches in a phylogeny contribute to ^q^D(A) or ^q^D(P), respectively. ^q^D(F) varies as a function of heterogeneity in functional uniqueness among species and has a maximum value equal to S when all species in an assemblage have equal proportional unique functional volumes. Functional trait values can be standardized in two ways [6]. First, all traits are standardized to a mean of zero and standard deviation of 1 (the z-transformation), which puts all traits on the same scale. Second, traits can be further standardized by the effect that each has on some ecological function. The second standardization is not trivial. To date, all analyses of functional diversity are based on the first standardization only. To distinguish these two types of standardization, Scheiner [6] referred to metrics based only on a z-transformation as a “trait-based metric” denoted as ^q^D(T) and used ^q^D(F) to refer to metrics that included standardizations based on functional effect or as a general reference for functional diversity.

Scheiner [6] conceived ^q^D(T) within the context of a set of points in functional space that could be enclosed by a convex surface and the metric is well behaved for such data. However, some assemblages have a single species that is functionally quite distinctive from all other species, or have multiple clusters of functionally similar species that are very distinct from other such clusters. In such cases, the behavior of ^q^D(T) is not clear. We explore some of these issues via exemplar datasets that were created to demonstrate maximal ^q^D(T), the insensitivity of ^q^D(T) to the range of functional values in an assemblage, effects of functional redundancy and highly distinct functional values on ^q^D(T) and how ^q^D(T) responds to randomly structured functional trait values (Table 3). For simplicity in these examples, the function of each species is described by a single trait value; however, the same general behaviors hold for functional diversity based on multiple traits.

**Table 3:** Functional trait values for 8 different traits for an assemblage of 10 species and resulting functional diversity values based on minimum functional distance, ^1^D(T), and total functional distance, ^1^D(T*).

Trait	A	B	C	D	E	F	G	H
Trait structure description	Even	Even-two groups	Redundant pairs	No variation	Distinct 1	Distinct 2	Random 1	Random 2
Species 1	1	1	1	5	1	1	23	74948
Species 2	2	2	1	5	2	2	60	14328
Species 3	3	3	2	5	3	3	81	34667
Species 4	4	4	2	5	4	4	95	60062
Species 5	5	5	3	5	5	5	68	41279
Species 6	6	101	3	5	6	6	7	95758
Species 7	7	102	4	5	7	7	78	51113
Species 8	8	103	4	5	8	8	3	7630
Species 9	9	104	5	5	9	9	74	43896
Species 10	10	105	5	5	100	1000	28	3526
V	6.61	0.38	0.00	0.00	6.63	6.36	3.37	5.31
^1^D(T)	10.00	10.00	1.00	1.00	1.65	1.07	8.80	8.06
FAD	24.22	21.41	23.85	0.00	14.37	12.82	23.55	23.78
^1^D(T*)	9.77	9.99	9.79	1.00	6.81	6.09	9.79	9.60

The structure of each trait was designed to demonstrate how differences in such structure affect estimates of functional diversity. V is the total unique functional volume of Scheiner [6] used to calculate ^1^D(T). FAD is the functional attribute diversity measure [26], which equals total functional distance (T) and is used to calculate ^1^D(T*). Note that functional values are z-transformed prior to diversity calculations.

If all species have the same minimum functional distances (Table 3, traits A and B), all species have equal proportional unique functional volumes (*f_i_*), resulting in a maximum ^q^D(T). In contrast, ^1^D(T) will have the minimum value of 1.0 for traits that have no unique functional volumes (traits C and D). ^1^D(T) will approach 1.0 for traits that have similar values for many species but for which one species is highly functionally distinct (traits E and F). Functional trait values that are randomly generated from a uniform distribution (traits G and H) will produce values of ^1^D(T) closer to S than to 1 because minimum functional distances between such values tend to be even. Similar to the effect of randomly generated abundances on ^q^D(A), randomly generated values with greater dispersion (trait H compared to trait G) produce less even distributions and lower values of ^q^D(T).

Because ^q^D(T) only uses information associated with the nearest functional neighbor for each species, it is not sensitive to some kinds of important functional variability among species. For example, traits A and B have the same ^q^D(T) value, despite A having completely evenly-spaced values and B having two groups of species with very different functional values. In addition, ^q^D(T) cannot distinguish between a trait with redundancy for many functional values (trait C) and a trait with no variation in function (trait D), because in each scenario, no species is functionally unique and functionally redundant species contribute nothing to ^q^D(T). Finally, ^q^D(T) is sensitive to highly distinct functional values (traits E and F) because it is based on proportional unique functional volumes, and highly distinct functional values can represent nearly all of the total unique functional volume (V), causing ^q^D(T) to approach 1 (Table 3).

### A functional dispersion metric based on Hill numbers

As an alternative to a metric based on minimum functional distances and the concept of unique functional volume, we propose a metric based on total pairwise functional distances and the concept of functional dispersion. Our new metric replaces the *minimum* functional distance (*d_i_* in [6]) with the *total* functional distance of each species to all other species:
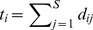
(3)where 
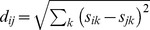
, the functional distance between the *i*th and *j*th species, and *s_ik_* is the standardized functional value of the *k*th trait for the *i*th species. Distance metrics other than Euclidian can be used. The total functional distance of all species is 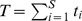, which is the equivalent to the functional attribute diversity measure (FAD of [26]). The proportional total functional distance of the *i*th species is 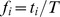. Using the Scheiner framework [6], functional diversity is:
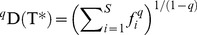
(4)


This new metric is similar to the previously proposed trait-based metric, ^q^D(T), except now functional diversity is measured as the total functional distance instead of as the unique functional volume. We use ^q^D(T*) to distinguish our new metric from the original definition for trait-based diversity. In addition, ^q^D(F*) indicates functional diversity based on total functional distance when standardizations based on contributions of traits to ecological function are employed. Our new metric reflects the functional dispersion of species, has a range from 1 to S and has maximum values when all species have equal total functional distances.

^q^D(T*) exhibits two noteworthy improvements compared to ^q^D(T). First, ^1^D(T*) can distinguish an even distribution of functional values associated with functionally redundant species (trait C in Table 3) from a distribution of invariant functional values (trait D) and gives intuitively consistent results (Table 3). Second, a single functionally distinct species will not cause values of ^1^D(T*) to approach 1.0, though values may decrease appreciably (traits A, E and F). More specifically, the only difference between functional data for traits A, E and F is the value for Species 10, which changes from 10 to 100 to 1000. These changes cause ^1^D(T) to decrease from 10 to 1.07, whereas ^1^D(T*) only decreases from 9.77 to 6.09. Nonetheless, a potentially undesirable behavior remains. Randomly generated trait values (traits G and H) resulted in highly even distributions of proportional total functional distances, indicating that this metric also is not able to distinguish between random patterns of variation in functional dispersion and maximal functional dispersion that results from limiting ecological similarity.

The replication principle is even more restrictive for functional data than for phylogenetic data. For functional data, replication holds only under two conditions: (1) the assemblages consist of unique species with redundant functional values (e.g. trait C in Table 3 split into two assemblages {1, 3, 5, 7, 9} and {2, 4, 6, 8, 10}) or (2) when all assemblages have identical total functional distances (*T*) and all pairs of assemblages have identical mean distances [16]. As with phylogenetic information, functional distinctiveness involves relational information. The additional restrictions occur because functional space does not have a uniquely defined root as does a cladogram.

### Simultaneous consideration of multiple traits

Although ^q^D(A) can be calculated based on different measures of abundance (e.g. number of individuals or frequency of occurrence) and many traits are combined to create the phylogenetic tree used to estimate ^q^D(P), each of these dimensions of biodiversity generally are calculated based on a single estimate of abundance or a single estimate of evolutionary divergence. In contrast, ^q^D(T) and ^q^D(T*) are formulated to be calculated based on multiple traits. This flexibility of ^q^D(T) and ^q^D(T*) requires careful consideration in the selection of functional traits because relationships between traits (e.g. multicollinearity) may affect diversity. In addition, calculating functional diversity separately for different functional components (e.g. diet, foraging method, body size, biomass production) may provide clues about the processes responsible for patterns of diversity. We explore the effects of combining traits with different patterns and relationships (i.e. correlated positively or negatively, or randomly associated) on ^q^D(T) and ^q^D(T*) and compared those to diversity based on only one of those functional traits (Table 4).

**Table 4:** Effects of using two traits to estimate functional diversity based on unique functional volume, ^1^D(T), or functional dispersion, ^1^D(T*).

^1^D(T) - unique functional volume										^1^D(T*) - functional dispersion									
2nd	Trait	Positive correlation between traits								2nd	Trait	Positive correlation between traits							
trait	description	Trait A	Trait B	Trait C	Trait D	Trait E	Trait F	Trait G	Trait H	trait	description	Trait A	Trait B	Trait C	Trait D	Trait E	Trait F	Trait G	Trait H
None		10.00	10.00	1.00	1.00	1.65	1.07	8.80	8.06	None		9.77	10.00	9.79	1.00	6.81	6.09	9.79	9.60
A	Even	0.00	0.00	*9.00*	*9.00*	0.05	0.57	−0.01	0.47	A	Even	−0.67	−0.24	−0.58	*8.10*	−0.04	0.65	−0.12	−0.29
B	Even - 2 groups	0.00	0.00	*1.05*	*9.00*	−0.64	−0.07	**−4.85**	**−4.54**	B	Even - 2 groups	−0.01	0.00	0.04	*9.00*	0.22	0.84	0.04	−0.05
C	Redundant pairs	0.00	**−7.95**	0.00	0.00	−0.43	0.12	−0.22	0.72	C	Redundant pairs	−0.56	−0.17	−0.59	*8.19*	0.10	0.77	−0.02	−0.18
D	Invariant	0.00	0.00	0.00	0.00	−0.64	−0.07	**−3.09**	**−3.19**	D	Invariant	**−**0.67	0.00	**−**0.59	0.00	**−4.65**	**−4.33**	−0.62	**−1.15**
E	Distinct 1	**−8.30**	**−8.99**	0.22	0.01	**−**0.64	**−**0.07	**−7.40**	**−6.30**	E	Distinct 1	**−2.99**	**−2.97**	**−2.88**	*1.16*	**−4.65**	**−4.07**	**−2.40**	**−2.82**
F	Distinct 2	**−8.36**	**−9.00**	0.20	0.00	**−**0.64	**−**0.07	**−7.47**	**−6.45**	F	Distinct 2	**−3.03**	**−3.08**	**−2.93**	0.76	**−4.79**	**−4.33**	**−2.47**	**−2.89**
G	Random 1	**−1.21**	**−6.05**	*7.57*	*4.70*	**−**0.25	0.26	−**3.10**	**−**0.83	G	Random 1	**−**0.09	**−**0.17	**−**0.02	*8.17*	0.58	*1.23*	**−**0.62	**−**0.73
H	Random 2	**−1.47**	**−6.49**	*7.77*	*3.87*	0.11	0.53	**−1.57**	**−3.19**	H	Random 2	**−**0.45	**−**0.45	**−**0.36	*7.45*	**−**0.03	0.63	**−**0.92	**−1.15**

2nd	Trait	Negative correlation between traits								2nd	Trait	Negative correlation between traits							
trait	description	Trait A	Trait B	Trait C	Trait D	Trait E	Trait F	Trait G	Trait H	trait	description	Trait A	Trait B	Trait C	Trait D	Trait E	Trait F	Trait G	Trait H
None		10.00	10.00	1.00	1.00	1.65	1.07	8.80	8.06	None		9.77	10.00	9.79	1.00	6.81	6.09	9.79	9.60
A	Even	0.00	0.00	*9.00*	*9.00*	0.05	0.57	**−**0.01	0.47	A	Even	**−**0.67	**−**0.24	**−**0.58	*8.10*	**−**0.04	0.65	**−**0.12	**−**0.29
B	Even - 2 groups	0.00	0.00	*1.05*	*9.00*	**−**0.64	**−**0.07	**−4.85**	**−4.54**	B	Even - 2 groups	**−**0.01	0.00	0.04	*9.00*	0.22	0.84	0.04	**−**0.05
C	Redundant pairs	0.00	**−7.95**	0.00	0.00	**−**0.43	0.12	**−**0.22	0.72	C	Redundant pairs	**−**0.56	**−**0.17	**−**0.59	*8.19*	0.10	0.77	**−**0.02	**−**0.18
D	Invariant	0.00	0.00	0.00	0.00	**−**0.64	**−**0.07	**−3.09**	**−3.19**	D	Invariant	**−**0.67	0.00	**−**0.59	0.00	**−4.65**	**−4.33**	**−**0.62	**−1.15**
E	Distinct 1	**−8.30**	**−8.99**	0.22	0.01	0.37	0.93	**−7.34**	**−6.25**	E	Distinct 1	**−2.99**	**−2.97**	**−2.88**	*1.16*	**−2.55**	**−1.96**	**−2.52**	**−2.68**
F	Distinct 2	**−8.36**	**−9.00**	0.20	0.00	0.36	0.93	**−7.40**	**−6.35**	F	Distinct 2	**−3.03**	**−3.08**	**−2.93**	0.76	**−2.69**	**−2.25**	**−2.59**	**−2.75**
G	Random 1	**−1.21**	**−6.05**	*7.57*	*4.70*	−0.19	0.32	−0.86	**−2.01**	G	Random 1	−0.09	−0.17	−0.02	*8.17*	0.45	*1.11*	−0.05	0.10
H	Random 2	**−1.47**	**−6.49**	*7.77*	*3.87*	0.16	0.63	**−2.75**	**−1.66**	H	Random 2	−0.45	−0.45	−0.36	*7.45*	0.11	0.76	−0.09	−0.10

2nd	Trait	Random association between traits								2nd	Trait	Random association between traits							
trait	description	Trait A	Trait B	Trait C	Trait D	Trait E	Trait F	Trait G	Trait H	trait	description	Trait A	Trait B	Trait C	Trait D	Trait E	Trait F	Trait G	Trait H
None		10.00	10.00	1.00	1.00	1.65	1.07	8.80	8.06	None		9.77	10.00	9.79	1.00	6.81	6.09	9.79	9.60
A	Even	**−1.58**	**−2.16**	*6.92*	*9.00*	0.25	0.56	−0.76	0.28	A	Even	−0.27	−0.26	−0.22	*8.10*	0.34	*1.35*	−0.14	−0.04
B	Even - 2 groups	**−2.16**	**−1.19**	*5.13*	*9.00*	−0.63	−0.07	**−2.45**	**−4.63**	B	Even - 2 groups	−0.03	−0.02	−0.06	*9.00*	0.34	0.86	−0.02	−0.07
C	Redundant pairs	**−2.08**	**−3.87**	*8.45*	0.00	−0.42	0.12	−0.15	−0.28	C	Redundant pairs	−0.20	−0.28	−0.17	*8.19*	0.10	0.77	−0.07	−0.16
D	Invariant	0.00	0.00	0.00	0.00	−0.64	−0.07	**−3.09**	**−3.19**	D	Invariant	−0.67	0.00	−0.59	0.00	**−4.65**	**−4.33**	**−**0.62	**−1.15**
E	Distinct 1	**−8.10**	**−8.98**	0.23	0.01	0.39	0.94	**−7.37**	**−6.22**	E	Distinct 1	**−2.62**	**−2.85**	**−2.87**	*1.16*	**−2.47**	**−1.93**	**−2.14**	**−2.67**
F	Distinct 2	**−8.36**	**−8.99**	0.20	0.00	0.36	0.93	**−7.60**	**−6.46**	F	Distinct 2	**−2.33**	**−3.05**	**−2.93**	0.76	**−2.65**	**−2.24**	**−2.10**	**−2.89**
G	Random 1	**−1.96**	**−3.65**	*7.65*	*4.70*	−0.23	0.13	**−2.63**	−0.73	G	Random 1	−0.12	−0.23	−0.07	*8.17*	0.84	*1.60*	−0.13	−0.10
H	Random 2	**−1.66**	**−6.57**	*6.78*	*3.87*	0.18	0.53	**−1.47**	**−1.85**	H	Random 2	−0.20	−0.47	−0.34	*7.45*	0.12	0.63	−0.29	−0.33

For each set of results, the top row is functional diversity values based on only one of the 8 trait structures from Table 3. Remaining rows indicate changes to functional diversity values that result from consideration of two traits for each possible combination of trait structures. For each combination of 2 traits, diversity was calculated with the traits being positively correlated (r = 1.0), negatively correlated (r = −1.0), or randomly associated. Negative and positive values indicate a reduction or increase in functional diversity, respectively. Functional diversity reductions or increases greater than 1.0 are in bold or italic text, respectively.

**Figure 2:**
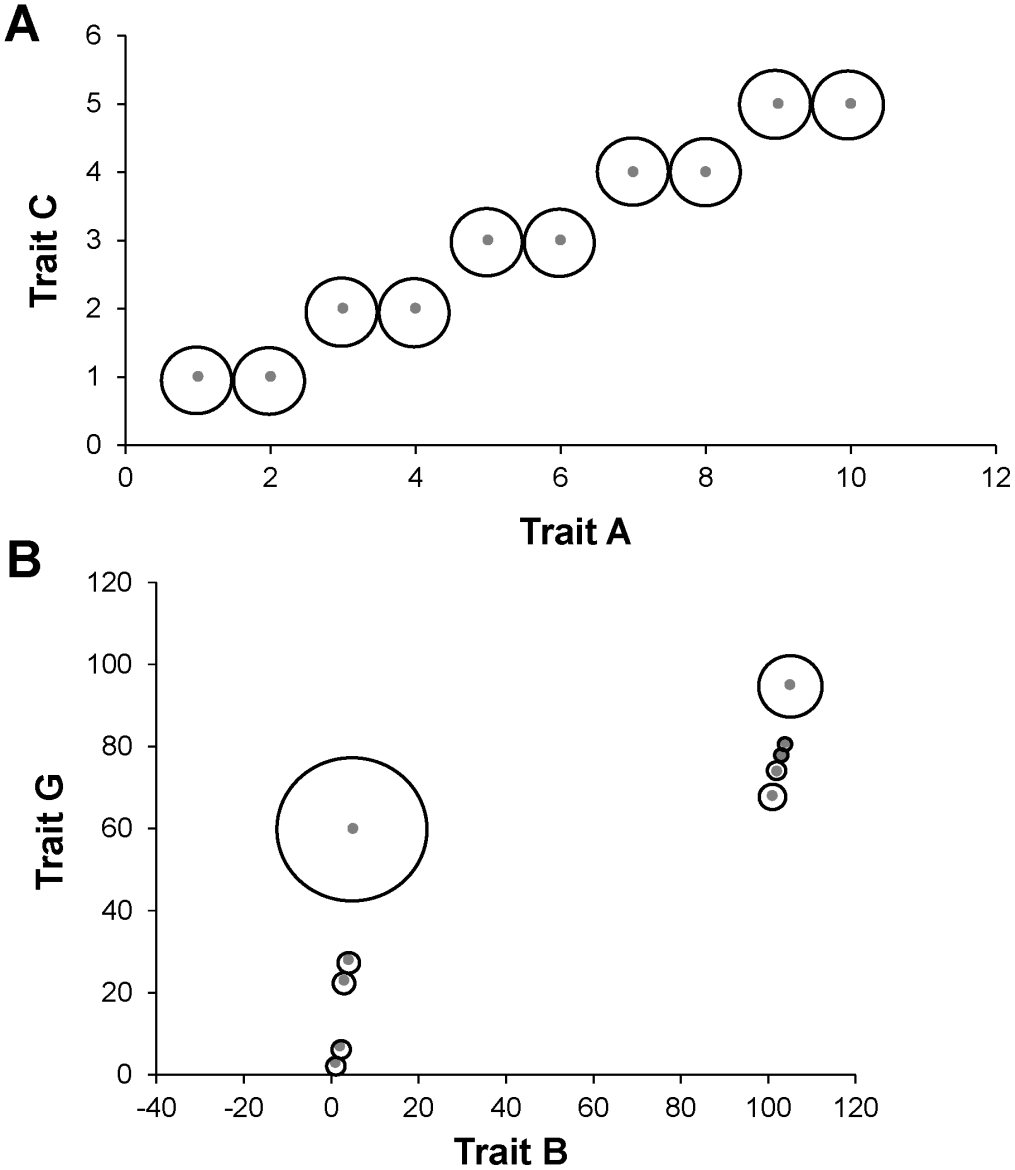
Examples of unique functional volumes in 2-dimensional trait space. A) An example in which all species have equal unique functional volumes that maximize functional diversity (traits A and C from Table 3). B) An example in which unique functional volumes differ greatly among species (traits B and G from Table 3). Gray dots represent locations of species in trait space and black circles represent unique functional volumes (i.e. radius equal to half the distance to the nearest neighbor in trait space). Axes are drawn such that units are equivalent and perfect circles represent associated unique functional volumes.

In general, the effect on ^1^D(T) or ^1^D(T*) of combining different patterns of trait values was not contingent on correlations between traits (Table 4). In some of these comparisons, functional diversity could not or was unlikely to increase or decrease because ^1^D(T) or ^1^D(T*) for a single trait was at or near the maximum or minimum value. ^1^D(T) increased appreciably (i.e. by more than 1.0) when a trait with no unique functional volume (traits C and D) was combined with randomly generated trait values or traits with even distributions. For example, ^1^D(T)  = 10.0 and 1.0 for traits A and B, respectively; however, consideration of both traits resulted in an equal distribution of distinct functional volume among all species (Figure 2A), essentially nullifying the effect of redundancy in the structure of trait B. Alternatively, ^1^D(T) declined appreciably when a trait with one distinct functional value (traits E and F) or with randomly generated values (traits G and H) was combined with a trait with perfectly even proportional unique functional volumes (traits A and B). For example, ^1^D(T)  = 10.0 and 8.8 for traits B and G, respectively; however, simultaneous consideration of these traits resulted in great heterogeneity among species in their unique functional volumes (Figure 2B) and greatly reduced ^1^D(T) to 3.95. Moreover, ^1^D(T) generally declined when at least one of the two traits had a random pattern (traits G and H). Interestingly, ^1^D(T) decreased appreciably if both traits were based on randomly generated values, especially when they were positively or negatively correlated (Table 4).

In contrast, the metric based on functional dispersion, ^1^D(T*), declined appreciably only when a trait with one distinct value (traits E and F) was combined with other trait structures (Table 4). Only for invariant trait D did ^1^D(T*) typically increase appreciably when combined with other trait structures. In general, the absolute differences between ^1^D(T) based on one trait and based on two traits was greater (mean change of 2.69) than for ^1^D(T*) (mean change of 1.67). This result is likely to be typical, as ^1^D(T) is based on the smallest functional distance of each species, which only gives weight to the single trait value with the least difference among species, whereas ^1^D(T*) is based on the total functional distance, which will give more equal weight to all trait values.

These results highlight the importance of trait selection when evaluating functional diversity, which has been highlighted previously as a general concern for estimating functional diversity [10], [27]–[29]. For ^1^D(T) and ^1^D(T*), particular care should be taken with respect to traits that are characterized by a single highly distinct value (traits E or F), as this greatly reduces estimates of functional diversity based on other traits (Table 4). In addition, care should be taken with regard to redundant (i.e. collinear) traits, as such relationships can result in a lower estimates of diversity than would occur for each redundant trait alone (e.g. diagonals of Table 4).

As with ^q^D(T), ^q^D(T*) can be combined with abundance and phylogenetic information in all possible combinations [^q^D(AT*), ^q^D(PT*) and ^q^D(APT*)]. We will examine such combined metrics in a future paper that explores patterns of diversity for bats from Costa Rica and Peru (Presley et al. in prep.).

### Comparisons with an alternative integrated approach

Anne Chao and collaborators [12], [16], [19] present an alternative method for combining abundance information with phylogenetic or with functional information within the Hill number framework. In their formulation, abundance and phylogenetic information are combined as:
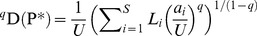
(5)where *L_i_* is the length of branch *i*, *a_i_* is the total abundance of the species descended from that branch, and *U* is the total evolutionary time interval. They also define a diversity measure in units of lineage length, 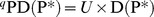 that can be linked to other metrics derived from Faith's PD. We use *^q^*D(P*) and *^q^*PD(P*) to indicate their metrics because their notation ^q^D(T), where T indicates time, can be confused with our usage of T to indicate the use of functional traits.

Abundance and functional information are combined as:
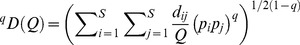
(6)where *Q* is Rao's quadratic entropy for functional diversity of an assemblage (typically called Rao's Q) and is calculated as:
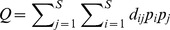
(7)


Again, there is an equivalently defined metric in units of functional distances (see Chiu and Chao [16] for details). The limitations described above concerning replication for phylogenetic and for functional data hold for each of these approaches.

The metrics *^q^*D(P*) and *^q^*D(Q) can be interpreted as the effective number of equally abundant species for a given total phylogenetic divergence and the effective number of equally abundant species for a given total functional distinctiveness, respectively. When all species are equally abundant, the metrics are equivalent to Faith's PD and to FAD, respectively. These are measures of abundance diversity weighed by total phylogenetic divergence or by total functional distinctiveness. In their approach, phylogenetic and functional diversity are measured as total divergence and distinctiveness, rather than as variation in divergence and distinctiveness as in the approach of Scheiner [6]. Their approach lacks the ability to provide measures of phylogenetic or functional diversity in units of effective numbers of species that are independent of abundance information except in the restricted case that all species are equally abundant. Nor does it permit the combining of phylogenetic and functional information. In contrast, our approach does not incorporate information about total phylogenetic depth or the total magnitude of functional distances. In our approach, those quantities are treated as independent in the same way that Hill diversity measured using abundances is independent of the total number of individuals (Table 1). Thus, these various metrics are based on different aspects of phylogenetic and functional diversity – variability versus total magnitude – resulting in numbers equivalents that represent different properties of communities. The challenge is to discover how to link all of these metrics to theories about the ecological and evolutionary processes that shape communities.

## Conclusions

Our framework is promising for the development of diversity metrics that have desirable properties and explicit units that facilitate ecologically meaningful comparisons among dimensions and among studies. In addition, our framework has two unique attributes that make it a powerful approach for considerations of biodiversity: (1) new metrics that measure different aspects of each dimension of biodiversity or new dimensions of biodiversity can be developed relatively easily and (2) it allows multiple aspects or dimensions of biodiversity to be integrated into a single measure. The flexibility and ease of developing new metrics for this framework is highlighted by the development of an alternative trait-based functional diversity metric, ^q^D(T*), based on total functional distance. Because ^q^D(T) and ^q^D(T*) measure different aspects of trait-based diversity associated with unique functional volumes and functional dispersion, respectively, each may have suitable uses for examining ecological or evolutionary processes responsible for patterns of diversity. Scheiner [6] detailed how to integrate information from multiple dimensions of biodiversity (i.e. abundance, phylogeny and function) into a single estimate. Such an approach can also be used to integrate different aspects of biodiversity within dimensions into a single measure. For example, for the functional dimension, one might wish to independently estimate diversity based on different niche axes (e.g. diet, foraging method, habitat use, masticatory mode, body size). These measures of *^q^*D(T*) could then be combined into a single value of functional diversity. Similarly, “importance” diversity could be separately calculated based on number of individuals, biomass and frequency of occurrence (following the traditional use of importance values developed in the vegetation literature [30], [31]), and then integrated into a single metric that simultaneously accounts for each of these ways of being biologically “important”. Such separate and integrated measures may be useful in testing theories about the myriad ecological and evolutionary processes responsible for patterns of biodiversity [8].
